# Cytotoxicity of antimicrobial wound irrigation solutions and antiseptics: an *in vitro* and *ex vivo* study

**DOI:** 10.3389/fmicb.2026.1945836

**Published:** 2026-09-21

**Authors:** Sophie Charlotte Liegenfeld, Nicolas Krueger, Anna-Lena Severing, Mandy Dittmer, Ewa K. Stuermer, Julian-Dario Rembe

**Affiliations:** 1 Department for Vascular Medicine, Translational Wound Research, University Heart and Vascular Center, University Medical Center Hamburg-Eppendorf (UKE), Hamburg, Germany; 2 MEDICE Arzneimittel Pütter GmbH & Co. KG, Iserlohn, Germany; 3 Department of Vascular and Endovascular Surgery, Medical Faculty and University Hospital Düsseldorf, Heinrich Heine University Düsseldorf (HHU), Düsseldorf, Germany

**Keywords:** antimicrobial, antiseptics, biocompatibility, chronic wound, cytotoxicity, wound infection, wound irrigation solution

## Abstract

**Introduction:**

Topical antiseptics must control wound bioburden without damaging cells required for tissue repair, yet cytocompatibility data obtained from isolated active compounds or monolayer exposure may not reflect finished formulations under clinically relevant conditions.

**Methods:**

This study compared the cytotoxic effects of six commercial antiseptics and wound-irrigation solutions across complementary *in-vitro* and *ex-vivo* models. An octenidine-based antiseptic, two polyhexamethylene biguanide formulations (0.1 and 0.04%) and three chlorine-based solutions (0.005% sodium hypochlorite/hypochlorous acid, 0.08% sodium hypochlorite, and 0.03% hypochlorous acid) were tested in HaCaT keratinocytes and CRL-2522 human dermal fibroblasts. Cell viability was assessed after 24 h using an ISO 10993-5-based assay and after 1, 5 and 15 min using an application-oriented exposure-and-washout assay. In addition, the cell viability and membrane integrity was assessed by a qualitative LIVE/DEAD staining. Repeated topical treatment was additionally evaluated over 15 days in a human full-thickness *ex-vivo* skin wound model.

**Results:**

Cytotoxicity was strongly dependent on formulation, exposure time, concentration, and cell type. Octenidine/Phenoxyethanol caused the greatest and most rapid loss of viability, with substantial effects after 1 min and the most pronounced epithelial disorganization *ex-vivo*. Both polyhexamethylene biguanide formulations showed cytotoxicity during continuous 24 h exposure and, despite better short-term tolerance, impaired epithelial continuity after repeated treatment. The 0.005% sodium hypochlorite/hypochlorous acid and 0.08% sodium hypochlorite formulations maintained viability above the 70% cytotoxicity threshold under all tested conditions and preserved epithelial architecture. The 0.03% hypochlorous acid formulation showed time-, concentration-and cell-specific toxicity, including marked keratinocyte damage after 15 min in LIVE/DEAD analysis.

**Discussion:**

Cytocompatibility cannot be inferred from antiseptic class or nominal active-agent concentration alone. Finished formulations should be evaluated across various clinically relevant exposure conditions and tissue-level models and product selection should balance antimicrobial efficacy with preservation of regenerative cells and tissue.

## Introduction

The increasing prevalence of chronic wounds poses a growing challenge for healthcare systems worldwide, both in terms of clinical outcomes and economic burden. Factors such as demographic shifts, the rising incidence of comorbidities (e.g., diabetes mellitus, peripheral arterial occlusive disease, and chronic venous insufficiency), and the widespread emergence of antimicrobial resistance contribute to the escalating incidence and complexity of wound infections (Gottrup et al., 2014; Kramer et al., 2018). Chronic wounds affect approximately 1 to 2% of the population in industrialized countries and are associated with an annual cost per patient ranging between 6,000€ and 10,000€, underscoring the need for optimized wound care strategies (Sen, 2025; Heyer et al., 2016).

Microbial colonization and infection represent a major impediment to normal wound healing. In particular, the formation of biofilms by pathogenic bacteria creates a hostile microenvironment that traps the wound in a prolonged inflammatory state (Dissemond et al., 2025; Percival, 2017; Schultz et al., 2017). This pathophysiological condition is marked by an excessive release of proteases and reactive oxygen species (ROS), the degradation of extracellular matrix components and impaired proliferation of key regenerative cell types, including keratinocytes and fibroblasts (Pastar et al., 2023; Raziyeva et al., 2021). Effective infection control, therefore, is a cornerstone of wound care.

In this context, antimicrobial wound irrigation solutions and topical antiseptics are widely employed as part of decolonization and biofilm management protocols. Compounds such as octenidine dihydrochloride (OCT), polyhexamethylene biguanide (PHMB), and hypochlorous acid (HOCl) have demonstrated broad-spectrum antimicrobial activity and are commonly used in both prophylactic and therapeutic settings (Dissemond et al., 2025; Kramer et al., 2018). However, similar to the systemic use of antibiotics, the application of these agents is associated with cytotoxic side effects that may compromise the viability and functionality of cells critical to tissue regeneration. Previous *in-vitro* and in-*vivo* studies have indicated a dose-dependent relationship between antiseptic efficacy and cytotoxicity (Hirsch et al., 2010; Ince et al., 2007; Kramer et al., 2018; Rembe et al., 2016; Severing et al., 2019). In particular, the deleterious effects of highly effective antiseptic agents on keratinocytes, fibroblasts, and endothelial cells have been demonstrated, revealing that their biocompatibility often falls short of clinical expectations (Hirsch et al., 2010; Ince et al., 2007). For example, PHMB-containing solutions and OCT-based preparations were found to significantly reduce cell viability *in-vitro*, especially under clinically common concentrations and relevant exposure times. However, these are antiseptics with proven high bactericidal activity and biofilm-reducing properties due to their residual effect and partial tissue penetration. Conversely, hypochlorous acid-based solutions have been proposed as more biocompatible alternatives due to their physiological mechanism of action, with HOCl in general being a naturally occurring effector molecule produced by neutrophils during the oxidative burst (Wang et al., 2007). However, the cytotoxic impact of HOCl- and NaOCl-containing products remains a subject of debate. Differences in manufacturing processes, stability, concentration, and physicochemical properties of the solutions can significantly affect their biocompatibility and safety (Severing et al., 2019). Generally, many marketed products have been insufficiently evaluated regarding their cytotoxic impact and contradicting results can be found in the literature.

Given the delicate balance between antimicrobial efficacy and cytocompatibility, a nuanced understanding of how pronounced the cytotoxic effect of antimicrobial agents for wound management actually is, needs to be developed. Our study thereby seeks to address this gap by assessing the cytotoxicity of widely used antiseptic wound irrigation solutions on keratinocyte and fibroblast cell lines *in-vitro* and *ex-vivo*. By applying standardized and modified cytotoxicity assays and histological evaluation, our aim is to provide evidence-based guidance for the rational selection of antimicrobial agents in wound care maximizing antimicrobial benefit while minimizing harm to regenerating tissue.

## Materials and methods

### In-vitro cytotoxicity testing

The cytotoxicity of octenidine-dihydrochloride (OCT - Octenisept®, Schülke & Mayr GmbH, Norderstedt, Germany), <0.08% sodium hypochlorite (0.08% NaOCl - Lavanox®, Serag Wiessner GmbH & Co KG, Naila, Germany), 0.005% NaOCl/HOCI (0.005% HOCl - Granudacyn®, Mölnlycke Health Care GmbH, Duesseldorf, Germany), 0.1% polyhexamethylene-biguanide (0.1% PHMB - Prontosan®, B. Braun SE, Melsungen, Germany), 0.1% polyhexamethylene-biguanide (0.04% PHMB - Serasept®, Serag Wiessner GmbH & Co KG, Naila, Germany), 0.03% hypochlorous acid (0.03% HOCl - Vashe®, Urgo Medical, North America) and phosphate-buffered saline (PBS - control) was assessed using human wound-relevant cell lines: immortalized human keratinocytes (HaCaT; Cell Lines Services GmbH, Eppelheim, Germany) and human dermal fibroblasts (BJ; ATCC® CRL-2522; American Type Culture Collection, Manassas, VA, USA). Cells were cultured in 96-well plates as adherent monolayers under standard conditions (37 °C, 5 %CO₂). PBS served as the negative control. No separate positive cytotoxicity control was included. Cytotoxicity was assessed using two experimental setups: (i) a norm-based assay based on DIN EN ISO 10993-5 with 24 h exposure (MTS-A) and (ii) an application-oriented assay without buffering to preserve product-specific pH effects, using clinically relevant exposure times of 1, 5, and 15 min (MTS-B). For the MTS-A, cells were seeded at 3,750 fibroblasts or 17,500 keratinocytes per well. Test-product concentrations were prepared in concentrated culture medium (10 × DMEM supplemented with FBS, L-glutamine and mg/ml NaHCO₃; GIBCO, Thermo Fisher Scientific, Waltham, MA, USA) and diluted in distilled water to final concentrations of 0% (control), 10, 25, 50, and 75% (v/v).

For the MTS-B, cells were seeded at higher densities (7,500 BJ or 30,000 HaCaT cells per well) to achieve 85–90% confluence by 24 h prior to treatment. Test-product concentrations were prepared as described for MTS-A. However, NaHCO₃ was omitted from the medium to avoid buffering of the test solutions and thereby preserve product-specific pH during exposure. Cells were exposed to the respective test solutions for 1, 5, or 15 min.

Following the respective exposure period, the test solutions were removed, cells were rinsed with PBS, and viability was determined using the MTS proliferation assay (Promega, Madison, WI, USA; 4 h of incubation; absorbance at 490 nm). Cell survival was expressed as a percentage relative to untreated controls (100%). In accordance with the cytotoxicity criterion defined in DIN EN ISO 10993-5, cell viability below 70% of the control was considered indicative of cytotoxicity. All experiment was performed in technical triplicates and independently repeated three times (*n* = 9).

### LIVE-DEAD staining

For qualitative assessment of cell viability, LIVE-DEAD staining was performed following the application-oriented exposure protocol (MTS-B; 15 min). After treatment, cells were gently rinsed with PBS and incubated with 500 μL FDA (Fluorescin diacetate, 20 μg/mL, Sigma-Aldrich, Merck KGaA, Darmstadt, Germany) and 40 μL PI (Propidium iodide, 50 μg/mL, Sigma-Aldrich, Merck KGaA, Darmstadt, Germany) per well for 3 min at room temperature. Excess stains were washed off with PBS and plates were air-dried. Cells were examined by fluorescence microscopy, at a wavelength of 481 nm (FDA) and 561 nm (PI) (ECLIPSE TS 100, Nikon Corporation, Tokyo, Japan) for viability (coloring), changes in morphology, including cell density, integrity, detachment, and compared with untreated controls. Viable cells were identified by green fluorescence, whereas cells with compromised membrane integrity were stained red. As dead cells may detach and be lost during handling, the red-stained population may underestimate the total number of non-viable cells.

### Assessment in a human *ex-vivo* full-thickness skin model

Human full-thickness skin obtained from abdominoplasties was used to establish an *ex-vivo* wound model (Strenge et al., 2024). Circular 8-mm skin samples containing a central 2-mm punch biopsy wound were placed in an air-liquid interface (“airlift”) culture system in 6-well plates, with culture medium (10x DMEM, supplemented with 10% FBS and 1% Penicillin–Streptomycin; GIBCO, Thermo Fisher Scientific, Waltham, MA, USA) reaching the upper dermis while the epidermis remained exposed to air. The models were maintained for 15 days. Every two days, wounds were treated topically with 10 μL of OCT, 0.08% NaOCl, 0.005% NaOCl/HOCl, 0.1% PHMB, 0.04% PHMB, 0.03% HOCl or 10 μL of PBS (control) for 15 min, followed by rinsing and medium exchange. At day 15, triplicate samples per condition were fixed in 4% paraformaldehyde, dehydrated, paraffin-embedded and sectioned (3 μm).

### Histological staining of an *ex-vivo* full-thickness skin model

Paraffin-embedded sections (3 μm) from *ex-vivo* full-thickness skin models were deparaffinized in xylene, rehydrated through a graded ethanol series and rinsed in distilled water before staining. Hematoxylin–eosin (H&E) staining was performed to assess general tissue morphology, epidermal architecture and re-epithelialization. Sections were incubated in hematoxylin for 5 min, washed in running tap water for 10 min to develop nuclear staining, rinsed in distilled water and counterstained with aqueous eosin for 10 min. Nuclei appeared blue and connective tissue reddish-pink.

Re-epithelialization was quantitatively assessed in H&E-stained sections obtained after 15 days of cultivation by measuring the length of the newly formed epithelial tongues extending from both wound margins toward the center of the wound. The lengths of both epithelial tongues were combined and related to the initial wound width. Re-epithelialization was expressed as a percentage of the initial wound width according to the following formula:

Re−epithelialization(%)=(total epithelial tongue length/initial wound width)×100

### Statistical analysis

Statistical analyses were performed using GraphPad Prism version 11.0.1 (GraphPad Software, Boston, MA, USA). Cell viability data are presented as mean ± standard error of the mean (SEM) from three independent experiments, each conducted with three technical replicates per condition. Relative cell viability was normalized to the phosphate-buffered saline (PBS) control, which was set to 1.0 (100%). Differences between treatment conditions and exposure times were assessed using two-way analysis of variance (ANOVA), followed by Dunnett’s multiple comparisons test to compare treatment groups with the PBS control. For the *ex vivo* model, quantitative re-epithelialization at day 15 was analyzed using one-way ANOVA, followed by Dunnett’s multiple comparisons test to compare each treatment group with the PBS control. Re-epithelialization data are presented as mean ± SEM from triplicate samples per condition. Statistical significance was defined as a two-sided *p* value <0.05. Significance levels are indicated as *p < 0.05, **p < 0.01, ***p < 0.001 and ****p < 0.0001.

## Results

### MTS cytotoxicity - negative control validation

For clarity, the antiseptic formulations OCT and PHMB are presented together in Figures 1, 2, whereas the hypochlorous acid (NaOCl/HOCl) formulations are presented in Figures 3, 4. PBS at all tested concentrations (10–75%) did not produce a consistent reduction in cell viability in either cell line, confirming its suitability as an inert control. Under 24-h exposure, PBS values remained close to the normalized reference in both CRL-2522 fibroblasts and HaCaT keratinocytes. Under the adapted short-term protocol (MTS-B), PBS controls also remained close to or above 100% viability after 1 and 5 min. After 15 min of exposure, PBS produced a reproducible increase in the MTS signal in fibroblasts, whereas this effect was not observed in HaCaTs. As MTS reduction reflects cellular metabolic activity rather than viable cell number directly, values above the untreated reference were interpreted as increased metabolic activity rather than enhanced cell survival or proliferation. In the NaHCO₃-free MTS-B setting, the phosphate-buffering capacity and ionic composition introduced by PBS may have contributed to this cell-specific response. However, as the underlying physicochemical and metabolic parameters were not assessed separately, the mechanism remains uncertain.

**Figure 1:**
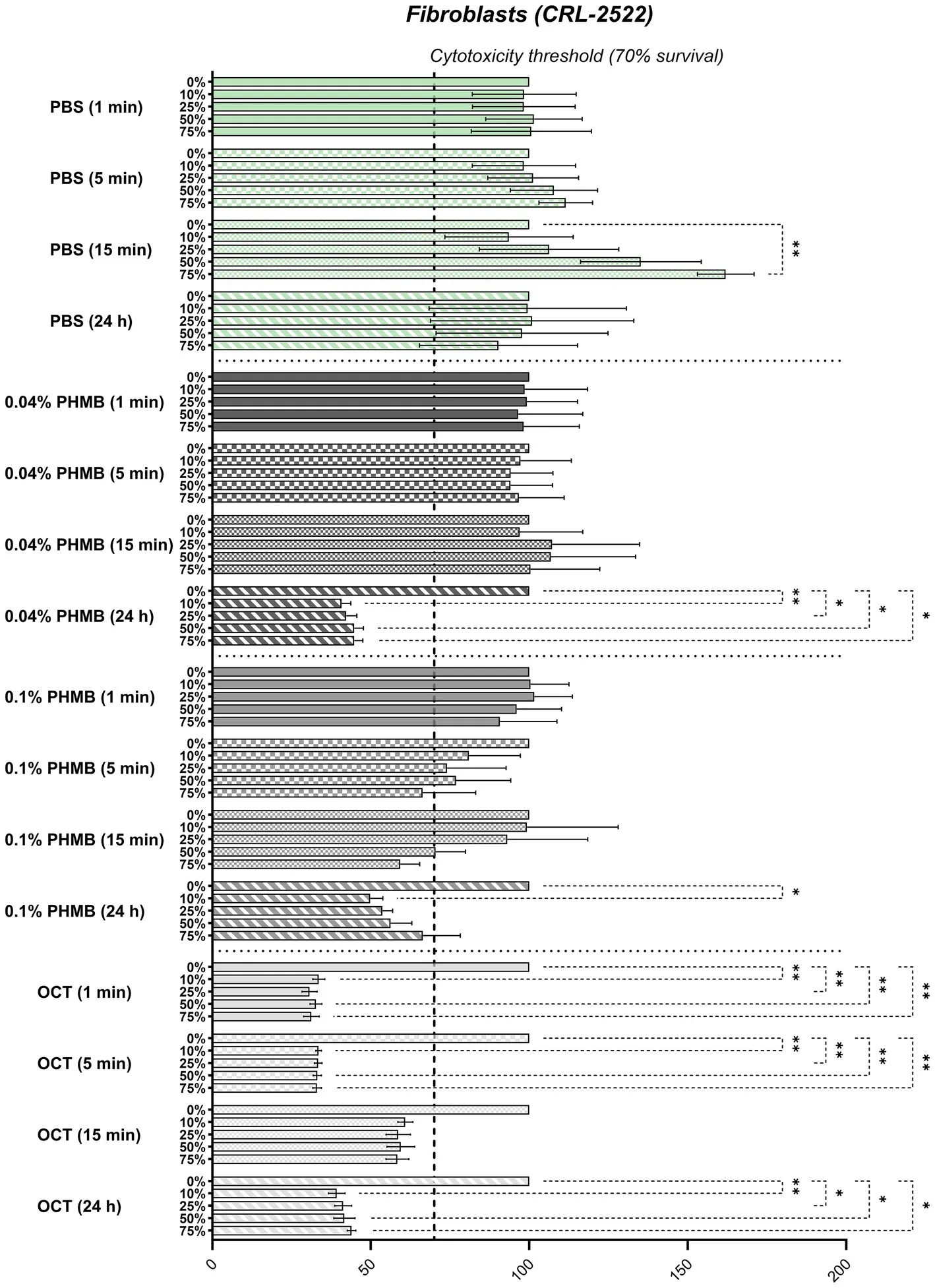
Cytotoxic effects of antiseptics on CRL-2522 fibroblasts. Relative cell viability of CRL-2522 fibroblasts following exposure to phosphate-buffered saline (PBS), polyhexamethylene biguanide (PHMB; 0.04 and 0.1%) and octenidine dihydrochloride (OCT) for 1 min, 5 min, 15 min (MTS-B) and 24 h (MTS-A), as determined by the MTS assay. Cell viability was normalized to the PBS control (set to 100% [1.0]). The dashed line indicates the threshold for cytotoxicity based on ISO 10993-5 (70% cell viability [0.7]). Data are presented as mean ± standard error of the mean (SEM) from *n* = 3 independent experiments with three technical replicates per independent experiment. Statistical comparisons were performed using two-way analysis of variance (ANOVA) with a Dunnett post-hoc test versus PBS control. *p < 0.05, **p < 0.01, **p < 0.001, ****p < 0.0001.

**Figure 2:**
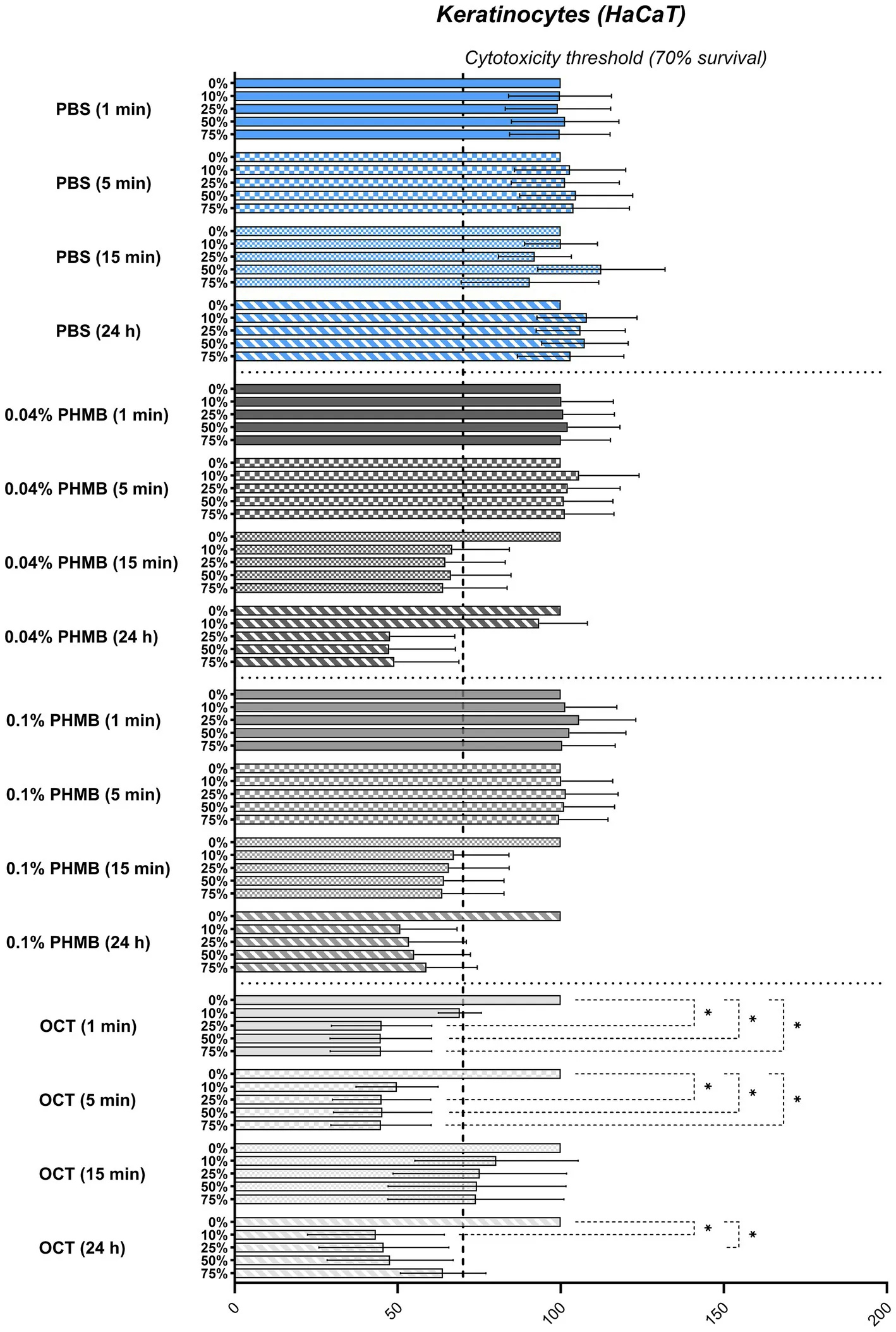
Cytotoxic effects of antiseptics on HaCaT keratinocytes. Relative cell viability of HaCaT keratinocytes following exposure to phosphate-buffered saline (PBS), polyhexamethylene biguanide (PHMB; 0.04 and 0.1%) and octenidine dihydrochloride (OCT) for 1 min, 5 min, 15 min (MTS-B) and 24 h (MTS-A), as determined by the MTS assay. Cell viability was normalized to the PBS control (set to 100% [1.0]). The dashed line indicates the threshold for cytotoxicity based on ISO 10993-5 (70% cell viability [0.7]). Data are presented as mean ± standard error of the mean (SEM) from *n* = 3 independent experiments with three technical replicates per independent experiment. Statistical comparisons were performed using two-way analysis of variance (ANOVA) with a Dunnett post-hoc test versus PBS control. *p < 0.05, **p < 0.01, **p < 0.001, ****p < 0.0001.

**Figure 3:**
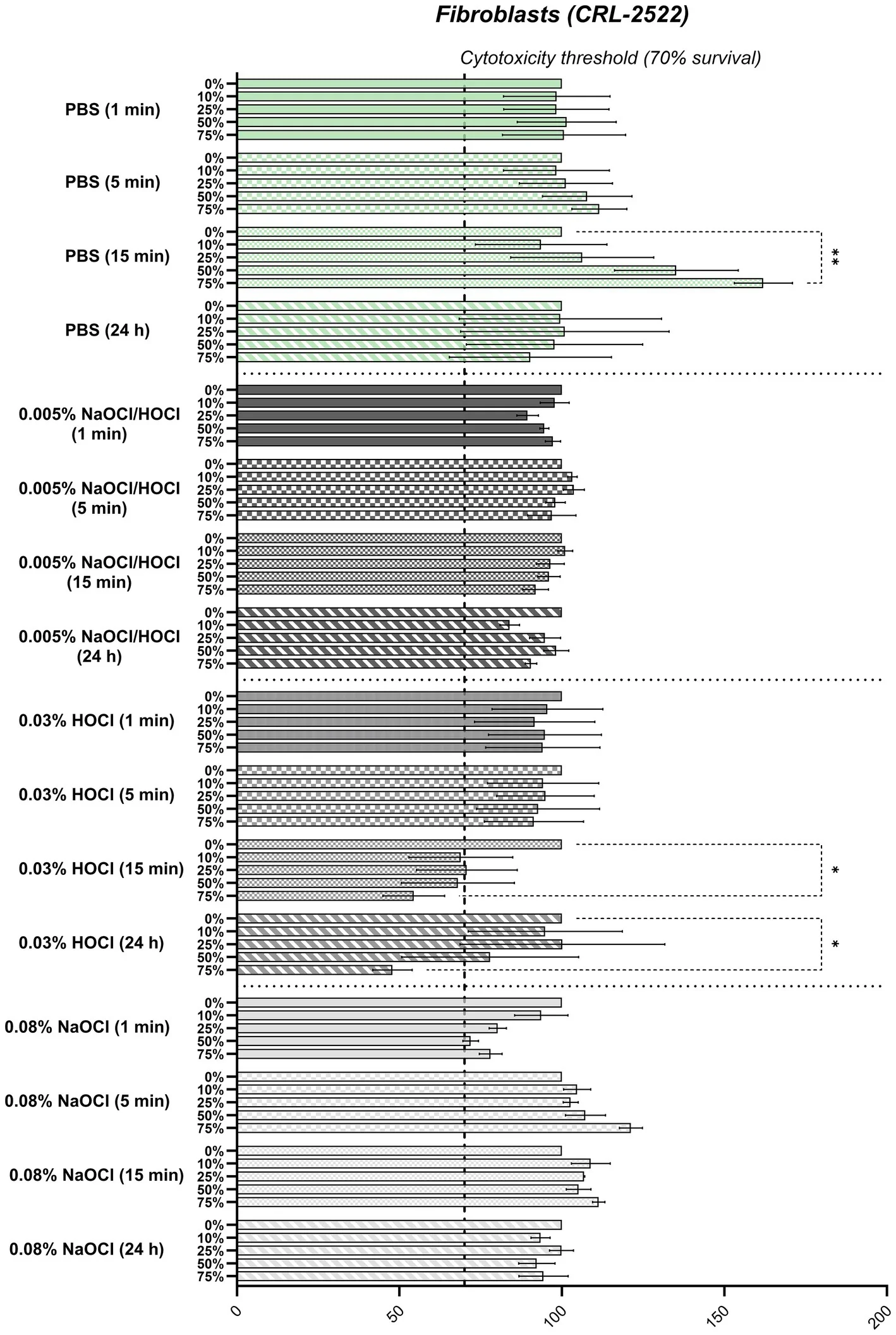
Cytotoxic effects of hypochlorous wound irrigation solutions on CRL-2522 fibroblasts. Relative cell viability of CRL-2522 fibroblasts following exposure to phosphate-buffered saline (PBS) and different hypochlorous acid wound irrigation solutions (0.005% NaOCl/HOCl, 0.03% HOCl and 0.08% NaOCl) for 1 min, 5 min, 15 min (MTS-B) and 24 h (MTS-A), as determined by the MTS assay. Cell viability was normalized to the PBS control (set to 100% [1.0]). The dashed line indicates the threshold for cytotoxicity according to ISO 10993-5 (70% cell viability [0.7]). Data are presented as mean ± standard error of the mean (SEM) from *n = 3 in*dependent experiments with three technical replicates per independent experiment. Statistical comparisons were performed using two-way analysis of variance (ANOVA) with a Dunnett post-hoc test versus PBS control. *p < 0.05, **p < 0.01, **p < 0.001, ****p < 0.0001.

**Figure 4:**
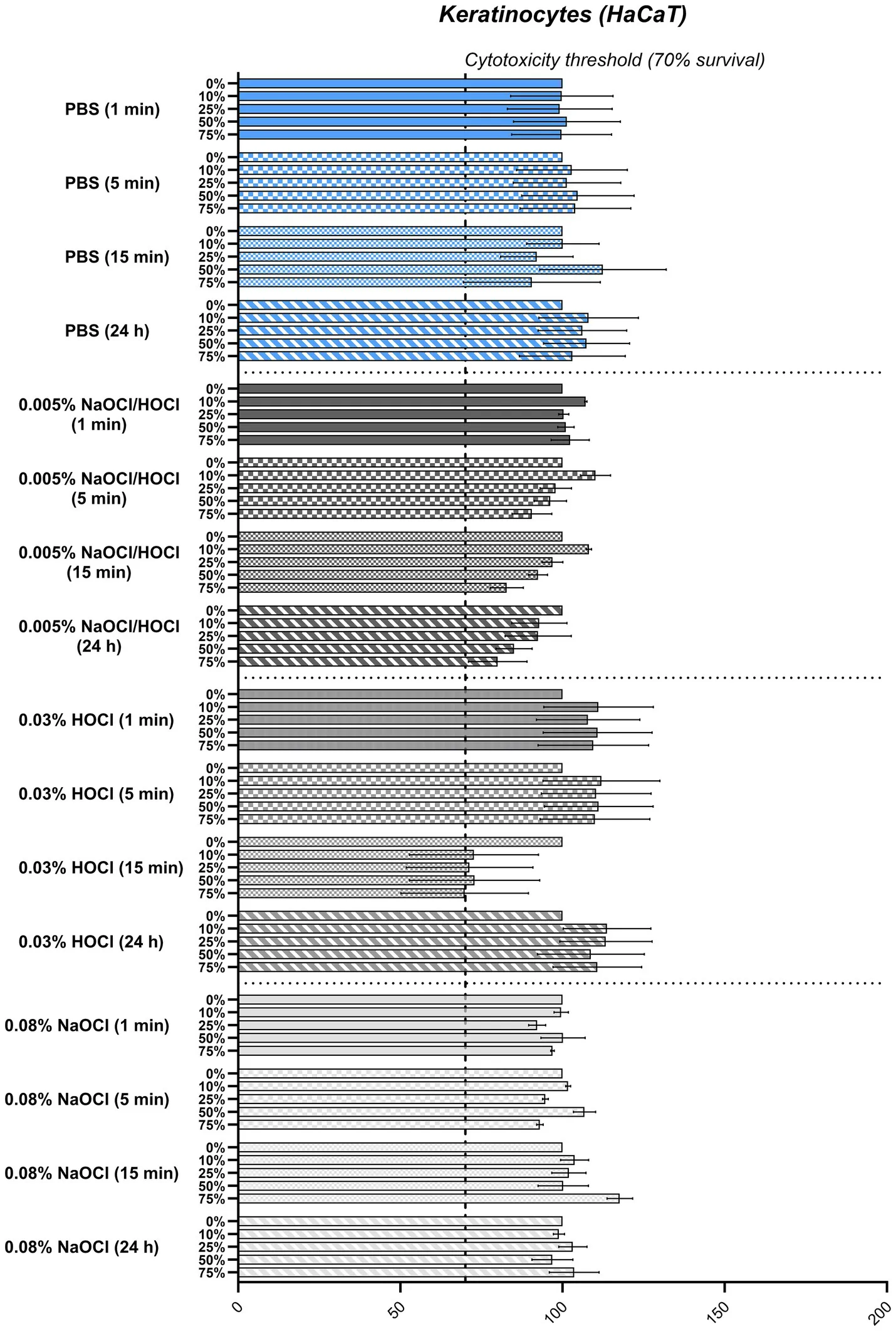
Cytotoxic effects of hypochlorous wound irrigation solutions on HaCaT keratinocytes. Relative cell viability of HaCaT keratinocytes following exposure to phosphate-buffered saline (PBS) and different hypochlorous acid wound irrigation solutions (0.005% NaOCl/HOCl, 0.03% HOCl, and 0.08% NaOCl) for 1 min, 5 min, 15 min (MTS-B) and 24 h (MTS-A), as determined by the MTS assay. Cell viability was normalized to the PBS control (set to 100% [1.0]). The dashed line indicates the threshold for cytotoxicity based on ISO 10993-5 (70% cell viability [0.7]). Data are presented as mean ± standard error of the mean (SEM) from *n* = 3 in dependent experiments with three technical replicates per independent experiment. Statistical comparisons were performed using two-way analysis of variance (ANOVA) with a Dunnett post-hoc test versus PBS control. *p < 0.05, **p < 0.01, **p < 0.001, ****p < 0.0001.

### MTS cytotoxicity of OCT

OCT demonstrated the strongest cytotoxic effects among the antiseptic formulations across both cell lines (Figures 1–4). Under 24-h exposure (MTS-A) in CRL-2522 fibroblasts, viability ranged from 39.2 to 43.9% across test-solution concentrations of 10–75%. In HaCaT keratinocytes, viability ranged from 43.3 to 63.9% over the same concentration range. The graphs indicate that substantial cytotoxicity was already present at the lowest tested concentration of 10% (Figures 1, 2).

OCT remained strongly cytotoxic under the brief exposure conditions of the MTS-B protocol. After 1 min in CRL-2522 cells, mean viability ranged from 30.7 to 33.6%, and after 5 min from 33.0 to 33.5%. At 15 min, viability increased to 58.4–60.9% but remained below the 70% cytotoxicity threshold (Figure 1). In HaCaT cells, 1-min exposure reduced viability to 44.8–69.1%, while 5-min exposure resulted in values of 44.9–49.8%. At 15 min, viability increased to 74.0–80.3%, indicating partial recovery above the cytotoxicity threshold (Figure 2). Taken together, OCT exhibits potent acute cytotoxicity that persists even after brief contact and washing.

### MTS cytotoxicity of 0.1 and 0.04% PHMB

Under 24-h exposure (MTS-A protocol), 0.1% PHMB reduced viability below the 70% cytotoxicity threshold in both cell lines (Figures 1, 2). In CRL-2522 cells, viability ranged from 49.8% at 10% test-solution concentration to 66.4% at 75%. In HaCaT cells, viability ranged from 50.8 to 58.8%.

Following 1 min of exposure in CRL-2522 cells, 0.1% PHMB showed little cytotoxicity, with viability values between 90.7 and 101.6%. After 5 min, viability decreased to 66.3–80.9%, and after 15 min to 59.3–99.2%, with considerable variability between concentrations and experiments (Figure 1). In HaCaT cells, viability remained near control levels after 1 min (100.5–105.6%) and 5 min (99.5–101.5%), but decreased to 63.7–67.2% after 15 min (Figure 2). These data indicate a contact-time-dependent cytotoxic effect of 0.1% PHMB under short-term exposure conditions.

The lower-concentrated 0.04% PHMB formulation also showed pronounced cytotoxicity under 24-h exposure. In CRL-2522 cells, viability ranged from 40.8 to 44.7% across the 10–75% test-solution concentration range, with little concentration dependence. In HaCaT cells, viability was 93.3% at 10% test-solution concentration but decreased to 47.6% at 25% and remained low at higher concentrations, reaching 49.0% at 75% (Figures 1–4).

In contrast, 0.04% PHMB was largely non-cytotoxic after brief exposure and washout. In CRL-2522 cells, viability ranged from 96.5 to 99.2% after 1 min and from 94.1 to 97.2% after 5 min. After 15 min, viability remained close to the control range at 96.9–107.2% (Figure 1). HaCaT keratinocytes also maintained viability above 100% after 1 and 5 min. After 15 min, however, viability decreased to 64.6–66.7%, indicating a cell-line-specific cytotoxic effect at the longer short-term exposure (Figure 2).

### Cytotoxicity of 0.005% NaOCl/HOCl

The 0.005% NaOCl/HOCl formulation showed very limited cytotoxicity in both cell lines across all tested exposure conditions (Figures 3, 4). In CRL-2522 cells, viability ranged from 88.8 to 97.2% after 1 min, 96.4 to 103.1% after 5 min, 91.3 to 100.5% after 15 min and 83.4 to 97.7% after 24 h. All values remained above the 70% cytotoxicity threshold and showed no consistent concentration-dependent decline (Figure 3).

HaCaT viability ranged from 100.0 to 106.7% after 1 min, 90.1 to 109.8% after 5 min and 82.3 to 107.7% after 15 min. Under 24-h exposure (MTS-A), viability decreased to 79.7–92.4%, with a modest concentration-dependent decline, but remained above the cytotoxicity threshold at all tested concentrations (Figure 4). No significant reduction relative to PBS was detected.

### Cytotoxicity of 0.03% HOCl

The cytotoxicity profile of 0.03% HOCl depended on exposure time and cell line (Figures 3, 4). In CRL-2522 cells, 1-min and 5-min exposure produced only minor reductions in viability, with values of 90.9–95.0% and 90.9–94.4%, respectively. After 15 min, viability decreased to 53.8–70.1%, placing all tested concentrations at or close to/below the cytotoxicity threshold. Under 24-h exposure (MTS-A), viability remained high at 10 and 25% test-solution concentration (94.3 and 99.5%), decreased to 77.4% at 50% and reached 47.3% at 75%.

HaCaT keratinocytes were less affected by 0.03% HOCl under most conditions. Viability remained above 100% after 1 min (107.4–110.7%), 5 min (109.5–111.5%) and 24 h (108.4–113.4%). After 15 min, however, viability decreased to 69.8–72.4%, close to the 70% cytotoxicity threshold across all tested concentrations (Figure 4).

### MTS cytotoxicity of 0.08% NaOCl

The 0.08% NaOCl formulation also showed a favorable cytotoxicity profile overall (Figures 3, 4). In CRL-2522 cells, viability after 1 min ranged from 71.3 to 93.0%, with the lowest value remaining just above the cytotoxicity threshold. Viability was preserved or increased after 5 min (102.2–120.8%) and 15 min (104.7–110.9%), and remained between 91.6 and 99.4% after 24 h. No consistent concentration-dependent decline was observed (Figure 3).

In HaCaT cells, viability remained above 90% under every exposure condition. Values ranged from 91.7 to 99.8% after 1 min, 92.6 to 106.4% after 5 min, 99.8 to 117.2% after 15 min and 96.5 to 103.3% after 24 h (Figure 4).

### Comparative summary of cytotoxic profiles

Among the antiseptic formulations, OCT showed the highest overall cytotoxicity, with marked reductions in viability after 1 and 5 min and after 24 h in both cell lines and only partial recovery after 15 min. Both PHMB formulations were strongly cytotoxic under 24-h exposure but substantially less harmful after brief contact and washout, particularly at 1 and 5 min. The effect of PHMB after 15 min differed between cell lines and formulations.

The NaOCl/HOCl formulations generally preserved cell viability more effectively than OCT and PHMB. Both 0.005% NaOCl/HOCl and 0.08% NaOCl maintained viability above the 70% threshold in CRL-2522 and HaCaT cells under all tested conditions. The 0.03% HOCl formulation showed a distinct time- and concentration-dependent reduction in CRL-2522 viability after 15 min and at higher concentrations under 24-h exposure. In HaCaT cells, this formulation remained non-cytotoxic except for values close to the threshold after 15 min. Overall, HaCaT keratinocytes showed greater tolerance than CRL-2522 fibroblasts to several formulations, most clearly under prolonged exposure to 0.03% HOCl and during the 15-min exposure to OCT.

### Visualization of formulation’s effect by LIVE/DEAD staining

The qualitative assessment of cell viability in CRL-2522 fibroblasts and HaCaT keratinocytes following 15 min of exposure was generally consistent with the results of the quantitative cytotoxicity assays. In addition to the distinction between viable cells, indicated by green fluorescence, and cells without membrane integrity, indicated by red fluorescence, a reduction in cell density relative to the PBS control was considered an additional indicator of cytotoxicity. This reduction was attributed, at least in part, to the detachment of non-viable cells from the culture surface and their subsequent removal during the washing steps. Therefore, the detected red cell fraction may underestimate the totality of non-viable cells.

Consistent with the quantitative cytotoxicity data, OCT caused a pronounced reduction in cell viability in both cell lines. Substantial cytotoxic effects were already observed at a concentration of 10%, and no clear concentration-dependent differences in cell survival were apparent. OCT treatment resulted not only in a marked reduction in cell density but also in PI-positive fluorescence in cells that remained attached to the culture surface, indicating loss of membrane integrity and cell death (Figure 5).

**Figure 5:**
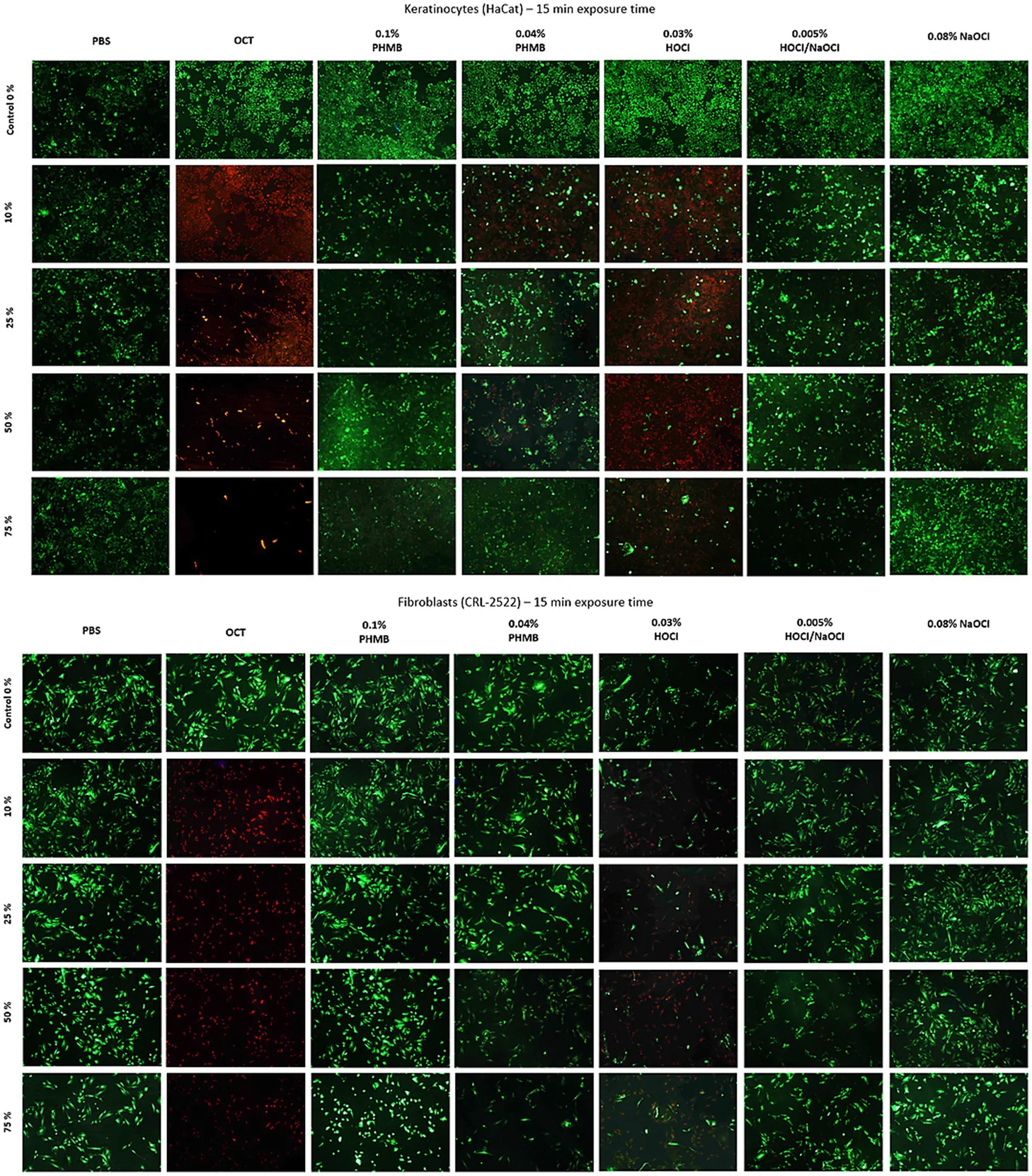
LIVE/DEAD staining of dose–response effects of antiseptics and wound irrigation solutions to CRL-2522 fibroblasts and HaCat keratinocytes on viability. Dose–response pictures, showing the viable cells in green and dead cells in red, following exposure to serial dilutions of polyhexamethylene biguanide (PHMB; 0.04 and 0.1%) and octenidine dihydrochloride (OCT) after 15 min of exposure. Test solutions were evaluated at dilution factors of 0, 10, 25, 50 and 75%.

By comparison, the solutions containing 0.04 and 0.1% PHMB produced less pronounced effects. In CRL-2522 fibroblasts, no clearly identifiable PI-positive cells were observed following treatment with either PHMB formulation. However, the 0.04% PHMB solution caused a reduction in cell density at test concentrations of 50 and 75%. Following exposure to the 0.1% PHMB solution, fibroblasts exhibited a more rounded morphology and lost their characteristic elongated phenotype, which may indicate cellular stress. In HaCaT keratinocytes, both PHMB-containing formulations caused a reduction in cell density that did not markedly increase at concentrations above 10%. A small number of PI-positive cells were also observed (Figure 5).

Among the wound irrigation solutions, the 0.005% NaOCl/HOCI and 0.08% NaOCl formulations showed little to no apparent effect on cell viability. Only the 0.005% NaOCl/HOCI solution caused a slight reduction in cell density at higher test concentrations in HaCaT keratinocytes. The 0.03% HOCI formulation also exhibited comparatively low to moderate cytotoxicity in CRL-2522 fibroblasts, consistent with the results of the preceding quantitative assays. After 15 min of exposure, a small number of PI-positive cells were observed at all tested concentrations. The overall cell count was also lower than that observed for the other solutions; however, it did not differ substantially from the PBS control. This suggests that the reduced cell density was not specifically attributable to the 0.03% HOCI formulation.

In HaCaT keratinocytes, by contrast, a pronounced reduction in cell density and a predominance of PI-positive cells were observed at concentrations of 10% and above (Figure 5). The cytotoxic effect observed by LIVE/DEAD staining appeared more pronounced than that indicated by the MTS assay, which showed an approximately 70% reduction in metabolic activity. This discrepancy may reflect differences between the assay principles, as the MTS assay measures residual metabolic activity, whereas FDA/PI staining provides a visual assessment of membrane integrity and cell viability at the single-cell level.

### Histological assessment of tissue architecture in the ex-vivo skin model

Histological evaluation of H&E-stained sections obtained after 15 days revealed distinct differences in epithelial architecture and tissue morphology between treatment groups (Figure 6A). PBS-treated controls displayed a continuous epithelial layer with preserved stratification and a well-organized tissue structure at the wound margins. Keratinocyte morphology appeared uniform, and epithelial cell layers remained cohesive and organized.

**Figure 6:**
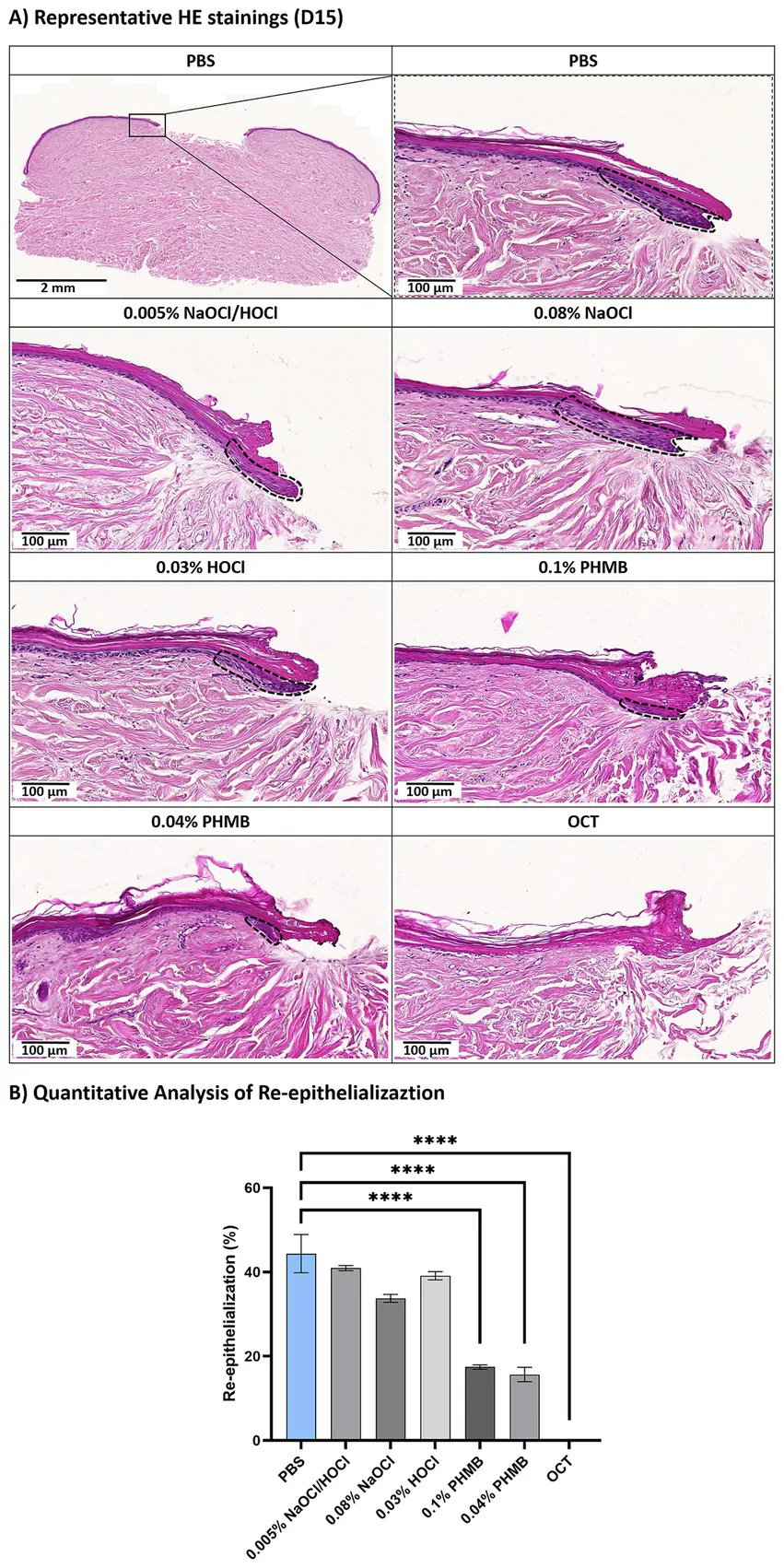
Differential effects of antiseptics and hypochlorous wound irrigation solutions on re-epithelialization in an *ex-vivo* human skin wound model. **(A)** Representative H&E-stained sections obtained 15 days after wounding demonstrate treatment-dependent differences in wound-edge morphology and epithelial regeneration. The overview image (upper left) depicts the ex-vivo wound model, with the boxed area indicating the wound edge shown at higher magnification. Dotted outlines indicate the newly formed epithelial tongues extending from the wound margins and illustrate the areas used for quantitative assessment of re-epithelialization. Treatment with hypochlorous acid–based wound irrigation solutions (0.005% NaOCl/HOCl, 0.03% HOCl and 0.08% NaOCl) was associated with well-organized, multilayered epithelial tongues, continuous epithelial fronts, preserved keratinocyte morphology, and maintained tissue architecture. In contrast, antiseptic treatment (0.1% PHMB, 0.04% PHMB and OCT) resulted in shortened and disorganized epithelial tongues, disrupted epithelial continuity, reduced cell cohesion, altered keratinocyte morphology, and impaired epithelial stratification. Scale bars: 2 mm (overview image) and 100 μm (high magnification images). **(B)** Quantitative analysis of re-epithelialization at day 15, expressed as the percentage of the initial wound width covered by the combined epithelial tongues extending from both wound margins. Data are presented as mean ± SEM from triplicate samples per condition. Statistical comparisons were performed using one-way ANOVA followed by Dunnett’s multiple comparisons test versus the PBS control. ****p < 0.0001.

Similar morphological features were observed following treatment with the hypochlorous acid-based wound irrigation solutions 0.005% NaOCl/HOCl, 0.08% NaOCl, and 0.03% HOCl. In all three groups, the epithelial compartment maintained a multilayered architecture with continuous epithelial fronts and preserved cell–cell cohesion. Keratinocyte morphology remained comparable to that observed in PBS-treated controls, and no obvious alterations in epithelial organization were apparent. Overall, epithelial stratification and tissue architecture were largely preserved.

In contrast, treatment with 0.1% PHMB, 0.04% PHMB and OCT was associated with more pronounced alterations in epithelial morphology. Compared with PBS controls and the hypochlorous acid-based solutions, these groups exhibited shortened epithelial fronts, reduced cellular cohesion, and disrupted epithelial continuity. Keratinocytes showed morphological alterations and an irregular arrangement within the epithelial compartment, accompanied by disrupted epithelial stratification and alterations in overall tissue architecture. The most pronounced histological changes were observed following OCT treatment, which exhibited the greatest degree of epithelial disorganization among all investigated treatments.

Quantitative assessment of re-epithelialization at day 15 supported these histological observations (Figure 6B). PBS-treated wounds showed a mean re-epithelialization of 44.4%. Comparable levels were observed following treatment with 0.005% NaOCl/HOCl (40.9%) and 0.03% HOCl (39.1%), while re-epithelialization following treatment with 0.08% NaOCl was 33.8%. In contrast, re-epithelialization was markedly reduced after treatment with 0.1% PHMB (17.4%) and 0.04% PHMB (15.7%), with both groups showing significantly lower re-epithelialization than the PBS control (both *p* < 0.0001). No measurable re-epithelialization was observed following OCT treatment (0%), which was likewise significantly lower than the PBS control (*p* < 0.0001). These quantitative findings corroborate the histological assessment, showing substantially impaired epithelial advancement after repeated PHMB and OCT treatment, whereas re-epithelialization was comparatively preserved after NaOCl/HOCl-based wound irrigation.

## Discussion

The present study demonstrates substantial differences in cytocompatibility between commonly used antimicrobial wound solutions. These differences depended on the formulation, exposure duration, test concentration, cell type, and experimental setting. Octenidine-dihydrochloride showed the strongest cytotoxic effects, with marked loss of fibroblast and keratinocyte viability after both short and prolonged exposure. The two PHMB-containing formulations produced pronounced cytotoxicity during continuous 24-h exposure but were better tolerated after brief contact and subsequent washout. The hypochlorous acid and sodium hypochlorite-based formulations generally showed the most favorable cytocompatibility profiles, although the 0.03% HOCl formulation caused distinct time-, concentration-, and cell-dependent effects. These findings were broadly supported by the LIVE/DEAD staining and the human *ex-vivo* skin model, in which the chlorine-based wound irrigation solutions preserved epithelial architecture more effectively than OCT and PHMB.

The clinical value of an antimicrobial wound solution depends on the balance between microbial reduction and preservation of host tissue generally termed biocompatibility (Kramer et al., 2018; Muller and Kramer, 2008). Keratinocytes and fibroblasts are central to re-epithelialization, extracellular matrix production, wound contraction, and tissue remodelling. Damage to these cell populations may therefore counteract the intended benefit of local antimicrobial treatment, particularly in chronic wounds in which regenerative capacity is already impaired. Previous comparative investigations have shown that antimicrobial potency and mammalian-cell toxicity do not necessarily follow the same ranking, leading to the concept of assessing antiseptics according to their overall biocompatibility rather than antimicrobial efficacy alone (Muller and Kramer, 2008; Severing et al., 2019). The present study extends this concept by comparing intact commercial formulations under both standardized and more complex application-oriented exposure conditions. Earlier work has likewise emphasized that cytotoxicity must be interpreted alongside antimicrobial activity rather than as an isolated product characteristic (Hirsch et al., 2010; Muller and Kramer, 2008).

OCT displayed the least favourable cytocompatibility profile in the present experiments. Viability was markedly reduced in both cell lines after only 1 min of exposure (Figure 1) indicating that substantial cytotoxicity was already reached at 10% of the commercial solution. Increasing the tested concentration produced little additional reduction in viability, suggesting that the cellular effect had approached a maximum within the investigated range. LIVE/DEAD staining (Figure 5) supported this interpretation by showing extensive loss of cell density and PI-positive cells among the remaining adherent populations. The *ex-vivo* findings further strengthened the biological relevance of the monolayer results. Here, repeated OCT treatment was associated with shortened epithelial fronts, loss of cellular cohesion, disturbed stratification and the most pronounced epithelial disorganization among the investigated products after 15 days of application (Figure 6). Additionally, a significantly reduced re-epithelialization was observed compared to control (*p* < 0.0001; Figure 6B).

The observed effects cannot be attributed to octenidine concentration alone. The tested product is a complete commercial formulation and its cellular effects may reflect the active antiseptic, other formulation components, or interactions between them. Commercial octenidine preparations contain phenoxyethanol in addition to octenidine (OCT/PE) (Besser et al., 2020). The current results therefore support conclusions about the tested formulation rather than isolated OCT as a chemical compound. They are in line with earlier studies performed solely in monolayer test systems and expand on these in terms of also demonstrating cytotoxic effects in more complex test systems such as *ex-vivo* models. Thereby they indicate that direct and repeated exposure of regenerating tissue to this product may carry a relevant cytotoxic burden. Its use should consequently remain indication-driven, with the anticipated antimicrobial benefit weighed against the potential effect on viable wound tissue. As a potent antiseptic OCT kills planktonic bacteria, fungi and viruses within one minute (Besser et al., 2020; Kramer et al., 2018; Muller and Kramer, 2008; Steinhauer et al., 2021) and is able to attack the biofilm matrix and the microorganisms embedded (Besser et al., 2020; Stuermer et al., 2021).

The effects of the PHMB-containing formulations were more dependent on exposure duration. Both products caused substantial cytotoxicity during the 24-h MTS-A protocol, whereas viability was largely preserved after 1 and, for several conditions, 5 min of exposure followed by washout. Cytotoxicity became more apparent with longer short-term exposure, particularly in HaCaT keratinocytes after 15 min (Figure 2). This pattern suggests that contact time may be at least as relevant as nominal PHMB concentration when estimating cellular tolerance under clinical conditions. When considering these, it should be noted that PHMB does reach its full antimicrobial effectiveness only after 10–15 min of exposure. Therefore, the 15-min cytotoxicity value may be considered as the clinically most relevant one. The results also show that a lower stated active-agent concentration does not necessarily predict better cytocompatibility. The 0.04% PHMB formulation caused strong cytotoxicity during 24-h exposure, in some conditions comparable with or greater than that observed for the 0.1% formulation. The tested products differ in their complete composition, including carrier solution, surfactants, ionic content, pH and other excipients. Prontosan®, for example, combines PHMB with a surfactant component, whereas the 0.04% formulation represents a different product matrix (Dissemond et al., 2025; Kramer et al., 2018; Wiegand et al., 2017). Direct ranking based only on the percentage of PHMB would therefore be misleading. The experimental unit in this study is the finished formulation at a defined dilution, not the active substance in isolation.

The *ex-vivo* results add an important dimension to the PHMB findings. Although PHMB produced less severe monolayer damage than OCT after short exposure, repeated treatment over 15 days was still associated with impaired epithelial continuity, shortened epithelial fronts, altered tissue organization and significant reduction in re-epithelialization compared control (*p* < 0.0001; Figure 6B). A single short exposure may therefore underestimate the cumulative consequences of repeated and prolonged treatment. This is clinically relevant because wound irrigation and antiseptic treatment are often repeated over several dressing changes and often prolonged unchecked based on overly confident biocompatibility claims derived from simple test systems. Previous experimental work comparing PHMB and OCT has also shown that effects observed in cell-based assays may translate into differences in wound contraction and epithelial repair in tissue or animal models (Hirsch et al., 2010; Kramer et al., 2004; Muller and Kramer, 2008; Salisbury et al., 2022). While PHMB is currently recognized as the most biocompatible antiseptic, it still needs to be used indication-focused, with caution and regularly re-evaluated over the treatment course.

The chlorine-based formulations showed a different overall pattern. The 0.005% HOCl/NaOCl and 0.08% NaOCl formulations maintained viability above the ISO 10993-5 cytotoxicity threshold (Figures 3, 4) under all investigated conditions. The *ex-vivo* tissue retained continuous epithelial fronts, multilayered organization and keratinocyte morphology comparable with PBS-treated controls (Figure 6). These results support the comparatively favourable tissue compatibility reported for stabilized low-concentration chlorine-based wound solutions (Severing et al., 2019; Wang et al., 2007). Stabilized HOCl has also been investigated as a wound-care agent because it combines oxidative antimicrobial activity with the potential for acceptable host-cell tolerance at appropriately controlled concentrations and physicochemical conditions (Severing et al., 2019, Wang et al., 2007). Nonetheless, in this case the products need to be investigated from the other angle of the antimicrobial efficacy/biocompatibility duality and previous work has shown, that in many cases, a comparably low antimicrobial effect is observed for these formulations (Rembe et al., 2020; Severing et al., 2022; Severing et al., 2019). In terms of a product which is deemed active against microorganisms, an improved biocompatibility should not be praised if the antimicrobial efficacy is lost.

The findings for 0.03% HOCl were more complex. Short exposure for 1 or 5 min caused little reduction in metabolic activity, but CRL-2522 viability decreased after 15 min and at higher test-solution concentrations during 24-h exposure. HaCaT viability remained high in the MTS assay under most conditions, with values near the cytotoxicity threshold after 15 min. LIVE/DEAD staining, however, showed a pronounced reduction in HaCaT cell density and a predominance of PI-positive cells after 15-min exposure. This result indicates that the cytocompatibility of chlorine-based solutions cannot be inferred from nominal chlorine concentration alone. The 0.08% formulation was better tolerated than the 0.03% formulation despite its higher stated concentration. Differences between chlorine-based products may arise from pH, the equilibrium between HOCl and hypochlorite ions, oxidation–reduction potential, free available chlorine, stability, buffering capacity, osmolality and additional formulation components (Severing et al., 2019, Wang et al., 2007). These properties determine both antimicrobial activity and interaction with mammalian cells. The three products should therefore be regarded as distinct formulations rather than interchangeable concentrations of the same active substance. The fact that HOCl is generated physiologically by activated neutrophils does not by itself establish the cytocompatibility of a commercial wound solution. Local concentration, exposure duration, chemical speciation and the surrounding biological matrix remain decisive, which is why more complex test systems such as the here demonstrated *ex-vivo* model are highly relevant for a comprehensive efficacy profiling of such formulations.

In order to address limiting discrepancies observed between the MTS and LIVE/DEAD results, their specific methodology should be considered. The MTS assay quantifies metabolic conversion within the population of cells remaining in the well. It does not directly measure cell number, membrane integrity or the proportion of detached cells. Cells that died and detached during exposure may have been removed during washing and therefore may not have contributed to the final MTS signal. Conversely, surviving cells with preserved or temporarily increased metabolic activity can produce values close to or above the control despite a reduced total cell number. The increased MTS signal observed specifically in CRL-2522 fibroblasts after 15-min PBS exposure should likewise be interpreted in this context. It may reflect a transient cell-specific metabolic response to the ionic and phosphate-buffered environment rather than increased fibroblast survival, particularly given the short exposure period and absence of a corresponding effect in HaCaT keratinocytes. FDA/PI staining evaluates membrane integrity at the single-cell level and allows simultaneous qualitative assessment of cell density and morphology. The more pronounced effect of 0.03% HOCl on HaCaT cells in the LIVE/DEAD assay may therefore reflect cell loss and membrane damage that were incompletely captured by metabolic measurement. The discrepancy supports the use of complementary viability methods rather than reliance on a single assay endpoint.

The comparison between MTS-A and MTS-B also illustrates the strong influence of experimental design. Continuous 24-h exposure according to the norm-based protocol represents a stringent hazard assessment but does not reproduce the usual clinical contact time of an irrigating solution followed by drainage, dilution or removal. The application-oriented protocol (MTS-B) more closely reflects short clinical exposures and demonstrated substantially better tolerance for PHMB and most chlorine-based products. At the same time, the absence of buffering in MTS-B exposes cells directly to product-specific pH and osmotic effects, some of which may be moderated by wound exudate, proteins, blood and tissue integrity *in-vivo*. Neither model should therefore be interpreted alone. Together, they describe the difference between intrinsic cytotoxic potential under prolonged exposure and cellular injury after brief product contact.

The human *ex-vivo* model strengthens the translational relevance of the study. It preserves native extracellular matrix, epidermal stratification, dermal structure and cell–cell interactions that are absent from monolayer cultures. Repeated treatment over 15 days also approximates cumulative product exposure more closely than a single cytotoxicity measurement. The concordance between preserved monolayer viability and maintained epithelial architecture for the 0.005 and 0.08% chlorine-based solutions is therefore notable. Likewise, the epithelial disruption observed after OCT and PHMB exposure supports the possibility that their *in-vitro* effects can influence tissue-level regeneration. Quantitative assessment of re-epithelialization at day 15 supported these histological observations, with significantly reduced re-epithelialization following PHMB and OCT treatment compared with PBS (*p* < 0.0001; Figure 6B), while no significant differences were observed for the NaOCl/HOCl-based wound irrigation solutions. The *ex-vivo* model used repeated topical treatment and histological assessment of human full-thickness skin, providing a bridge between isolated cell assays and clinical wounds. These results have practical implications, although they do not establish direct clinical superiority of one product. Nonetheless, they emphasize clearly, that cytotoxicity represents one side of the therapeutic balance, also beyond monolayer model results. The present study however did not provide a paired quantitative comparison of antimicrobial or biofilm reduction and host-tissue effects for each formulation, as these investigations have been performed in multiple previous studies of the authors (Besser et al., 2020; Rembe et al., 2016; Rembe et al., 2020; Severing et al., 2019; Stuermer et al., 2021). The here presented results combined with earlier investigations highlight, that a highly cytocompatible product may be insufficient against a mature biofilm or even in simple antimicrobial baseline tests (Kapalschinski et al., 2017; Radischat et al., 2020; Rembe et al., 2020; Schedler et al., 2017; Severing et al., 2022). Whereas a more cytotoxic antiseptic may still be justified for a heavily contaminated or clinically infected wound when used for a defined period, as infection-mediated tissue-damage takes precedence over cytotoxicity-mediated negative effects. Product selection should therefore account for the clinical indication, microbial burden, wound-healing phase, viable tissue exposure, intended contact time and need for repeated application. The current findings support limiting unnecessary or prolonged exposure of both antiseptics as well as antimicrobial wound irrigation solutions and selecting the shortest effective regimen compatible with adequate antimicrobial control.

While the combination of multiple test scenarios and methodologies including translational methods improve the results interpretability, naturally, some limitations to the study design and therefore interpretation and extrapolation of results need to be noted. HaCaT and CRL-2522 cells are established cell lines and do not fully represent primary cells from chronic wounds, which may differ in senescence, inflammatory activation, oxidative stress and treatment susceptibility. The MTS assay measures metabolic activity rather than wound closure, migration, proliferation or matrix synthesis, therefore only painting a limited picture of the cellular metabolism as a whole. Physicochemical parameters such as pH, osmolality, oxidation–reduction potential, free available chlorine and chemical stability were not measured alongside cytotoxicity in this study, limiting mechanistic interpretation of differences between formulations.

Future studies should, as a standard, combine cytotoxicity and antimicrobial efficacy within the same experimental framework. Such a framework should move closer towards clinically relevant variables and testing-platforms and include planktonic as well as biofilm-associated organisms and methods, clinically relevant organic loads (e.g., protein-rich wound-like media), repetitive treatment and quantitative tissue regeneration endpoints with translational models such as the ex-vivo model or organotypic models mimicking the tissues architecture. Primary wound-derived keratinocytes and fibroblasts, immune-competent tissue models (e.g., 3D wound models) and infected *ex-vivo* wound models would further improve clinical translation.

## Conclusion

Overall, the tested wound irrigation solutions showed clearly distinct cytotoxicity profiles. The highly effective antiseptic OCT caused rapid and sustained cellular damage and the most pronounced disruption of epithelial architecture *ex-vivo*. The also antiseptic PHMB-containing formulations were better tolerated after brief exposure but showed substantial cytotoxicity during prolonged or repeated contact. The NaOCl/HOCl wound irrigation solutions generally provided the best preservation of fibroblast and keratinocyte viability and maintained epithelial organization in the *ex-vivo* model, however the positive biocompatibility needs to be overall evaluated in light of previously reported inferior antimicrobial efficacy. Product selection should therefore be based on the complete formulation, intended exposure conditions and the balance between antimicrobial need and preservation of regenerative tissue.

## Data Availability

The raw data supporting the conclusions of this article will be made available by the authors, without undue reservation.
